# Autochthonous dengue outbreak in Marche Region, Central Italy, August to October 2024

**DOI:** 10.2807/1560-7917.ES.2024.29.47.2400713

**Published:** 2024-11-21

**Authors:** Chiara Sacco, Augusto Liverani, Giulietta Venturi, Stefano Gavaudan, Flavia Riccardo, Giovanna Salvoni, Claudia Fortuna, Katia Marinelli, Giulia Marsili, Alessia Pesaresi, Carla Molina Grané, Irene Mercuri, Mattia Manica, Sara Caucci, Daniela Morelli, Lolita Sebastianelli, Maurilia Marcacci, Federica Ferraro, Marco Di Luca, Ilaria Pascucci, Christina Merakou, Anna Duranti, Ilaria Pati, Letizia Lombardini, Daniel Fiacchini, Giorgio Filipponi, Francesco Maraglino, Anna Teresa Palamara, Piero Poletti, Patrizio Pezzotti, Fabio Filippetti, Stefano Merler, Martina Del Manso, Stefano Menzo, Marche dengue outbreak group, Marche dengue outbreak group, Antonello Amendola, Elisa Antognini, Claudio Argentini, Antonino Bella, Cristina Canonico, Valentina Curini, Valeria Di Lollo, Francesca Diotallevi, Raffaele Donadio, Cristiano Fiorentini, Alessandra Galluzzi, Elisa Mancuso, Luana Fiorella Mincarelli, Lucia Mosca, Miruna E. Rosu, Barbara Secondini, Francesco Severini, Simonetta Pupella, Angela Taraschi, Federica Tontarelli, Luciano Toma, Anna Valenza

**Affiliations:** 1 Department of Infectious Diseases, Istituto Superiore di Sanità, Rome, Italy; 2 ECDC Fellowship Programme, Field Epidemiology path (EPIET), European Centre for Disease Prevention and Control (ECDC), Stockholm, Sweden; 3 Pesaro Urbino Local Health Unit, Pesaro and Urbino, Pesaro, Italy; 4 Istituto Zooprofilattico Sperimentale Umbria e Marche “T. Rosati”, Ancona, Italy; 5 Centro Regionale Sangue-Regione Marche, AOU delle Marche, Ancona, Italy; 6 Department of Biomedical Sciences and Public Health, Università Politecnica delle Marche, Ancona, Italy; 7 Center for Health Emergencies, Fondazione Bruno Kessler, Trento, Italy; 8 Istituto Zooprofilattico Sperimentale dell'Abruzzo e del Molise “G. Caporale”, Teramo, Italy; 9 ARS Marche, Ancona, Italy; 10 Italian Ministry of Health, Rome, Italy; 11 National Blood Centre, Istituto Superiore di Sanità, Rome, Italy; 12 Italian National Transplant Centre, Istituto Superiore di Sanità, Rome, Italy; 13 Regione Marche – GORES Ancona, Ancona, Italy; 14 The members of the Marche dengue outbreak group are listed under Collaborators

**Keywords:** Italy, Dengue virus type 2, autochthonous transmission, *Aedes albopictus*

## Abstract

Between August and 28 October 2024, 199 autochthonous cases of dengue virus serotype 2 were notified in the city of Fano, central Italy. We describe the ongoing epidemiological and microbiological investigation and public health measures implemented to contain the outbreak. The high transmissibility and the extension of the outbreak suggest that dengue should be expected in temperate regions during favourable seasons, highlighting the need for heightened awareness among healthcare providers and the public to ensure timely detection and response.

In Europe, dengue viruses, transmitted by *Aedes albopictus* mosquitoes, are primarily associated with infections acquired in endemic countries. Local transmission remains rare, with only sporadic or small-scale outbreaks documented [1-6]. In 2020, Italy identified its first local transmission event of dengue virus with 11 notified cases [7]. Here, we describe an ongoing outbreak of 199 autochthonous cases in Fano, a small coastal city with ca 61,000 inhabitants, in Marche Region, central Italy, from August to early October 2024.

## Initial detection and epidemiological investigation

In early September, at least six individuals with fever (> 39°C), rash on hands and feet, nausea or vomiting and diarrhoea, independently visited the emergency department of the local hospital in Fano. Samples from these patients were sent to the Regional Reference Laboratory (RRL), Virology Unit of Azienda Ospedaliera Universitaria delle Marche, Ancona. Based on the symptoms, the physicians requested tests for hepatitis E, enterovirus, parvovirus B19 and dengue virus.

The emergency department informed the Local Health Authority (LHA) of Pesaro and Urbino, which initiated an epidemiological investigation, focusing on travel history (including areas endemic for arbovirus and other international travel) and food consumption. However, no remarkable findings emerged, and the patient samples were thus not initially tested for dengue.

On 11 September, the index case tested positive for dengue virus serotype 2 (DENV-2) by real-time PCR; the following day, the diagnosis was confirmed for the other five cases. Further investigations into potential common exposures in the 14 days preceding symptom onset revealed that all six were living (or had been visiting) in the same neighbourhood in Fano.

On 12 September, the LHA alerted all emergency departments and general practitioners in Fano to consider dengue as a possible diagnosis, even in patients without travel history to endemic countries but presenting with dengue-like symptoms. Cases were also identified retrospectively. Samples were forwarded to the RRL for testing.

We used the case definition criteria of the Italian National Plan for Prevention, Surveillance and Response to Arboviruses (2020–2025) [8]: a probable case is defined as an individual exhibiting symptoms consistent with dengue (fever > 39°C, nausea or vomiting, rash, aches and pains, retro-ocular pain) with a positive serology for IgM antibodies. A confirmed case requires laboratory confirmation, which may involve virus isolation, detection of viral RNA or dengue viral antigen (NS1) or the presence of dengue-specific IgM antibodies in a single serum sample and confirmation by neutralisation or seroconversion or four-fold antibody titre increase of dengue-specific antibodies in paired serum samples.

By 28 October, 138 confirmed and 61 probable cases of DENV-2 were notified. Although a comprehensive epidemiological investigation was carried out, we could not identify any case with links to international travel. The demographic characteristics and clinical data of the cases are shown in Table.

**Table:** Demographic and clinical data of probable and confirmed autochthonous dengue cases, Fano, Marche Region, Italy, August–October 2024 (n = 199)

Variables	n	%	Hospitalisation					
			Yes (n = 48)		No (n = 137)		Not indicated (n = 14)	
			n	%	n	%	n	%
Sex								
Female	97	48.7	21	43.8	68	50.4	7	NA
Male	102	51.3	27	56.2	67	49.6	7	NA
Symptoms								
Fever	179	89.9	44	91.7	130	96.3	3	NA
Asthenia	164	82.4	39	81.2	121	89.6	3	NA
Arthralgia	113	56.8	23	47.9	86	63.7	3	NA
Rash	65	32.7	23	47.9	42	31.1	0	NA
Nausea or vomiting	41	20.6	16	33.3	24	17.8	1	NA
Retro-ocular pain	8	4.0	2	4.2	6	4.4	0	NA
Median age (IQR)	n	Range	n	Range	n	Range	n	Range
Age (years)	62	44–74	60	37–74	63	44–74	64	47–73

IQR: interquartile range; NA: not applicable, percentages not given due to the small number of cases.

## Transmission dynamics, reporting delays and geographic distribution

**Figure 1:**
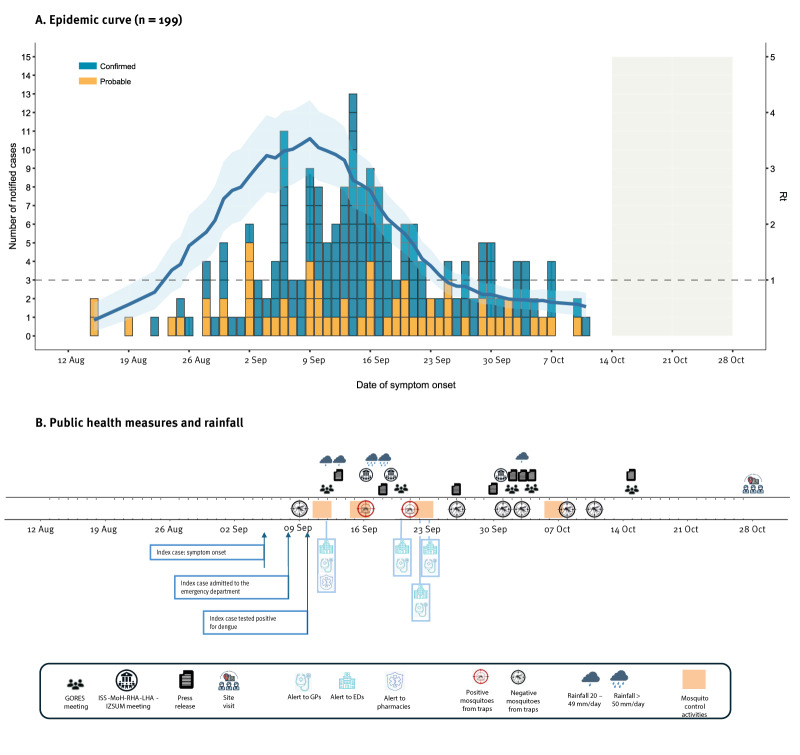
Epidemic curve of probable and confirmed autochthonous dengue cases and public health measures taken to contain a dengue outbreak, Fano, Italy, August–October 2024 (n = 199)

ED: emergency department; GORES: Regional Operational Group for Health Emergencies; GPs: general practitioners; ISS: Istituto Superiore di Sanità; IZSUM: Istituto Zooprofilattico Sperimentale Umbria-Marche; LHA: Local Health Authority; MoH: Ministry of Health; RHA: Regional Health Authority; Rt: net reproduction number.

Figure 1A shows the epidemic curve by symptom onset date. The symptoms of the first cases started in mid-August, with the peak of cases occurring in mid-September, just after the diagnosis of the index case. The symptoms of the latest notified case between August and 28 October started on 11 October. As this is an ongoing outbreak, data are not consolidated, and cases notified in the latest weeks are likely underestimated due to diagnostic delays.

Panel A. Confirmed (blue) and probable (yellow) dengue cases by date of symptom onset are presented. The area shaded in light blue indicates the R_t_ and the 95% confidence intervals. The grey shaded area indicates the window of the possible reporting delay.

We estimated reproduction numbers using a previously described method [9]. The basic reproduction number (R_0_) was estimated at 2.66 (95% confidence interval (CI): 2.08–3.31). The net reproduction number (R_t_) peaked at 3.53 (95% CI: 2.91–4.22) at the time of outbreak detection and implementation of vector control. The R_t_ dropped below the epidemic threshold around 25 September (Figure 1A). We obtained similar results when restricting the analysis only to confirmed cases. The average reporting delay across the study period was 15 days (2.5th–97.5th percentiles: 3–51.2), ranging from 23.4 days (2.5th–97.5th percentiles: 3–55.2 days) before the outbreak detection to 10 days (2.5th–97.5th percentiles: 2.1–26 days) thereafter. Details on the methods including assumptions for estimating the reproduction numbers and the reporting delay are presented in the Supplementary Material.

**Figure 2:**
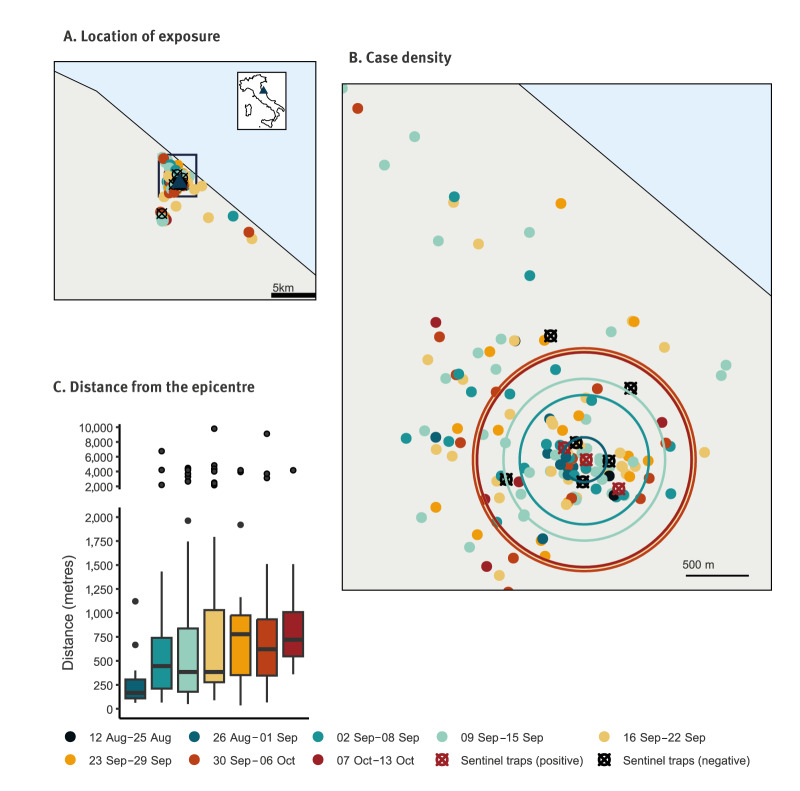
Geographic distribution of probable and confirmed autochthonous dengue cases, by week of symptom onset, Fano, Italy, August–October 2024 (n = 199)

Figure 2 shows the geographic distribution of likely exposure of the cases, by week of symptom onset. First cases (12 August–25 September) were highly localised, with a median distance of 169 m (interquartile range (IQR): 100–199 m) from the outbreak epicentre, defined as the centroid of the geolocated cases over these 2 weeks. However, the median distance started to increase after 2 September reaching 802 m (IQR: 362–1,071 m) by 29 September.

Panel A. The geographic distribution of dengue cases by exposure location across the outbreak area is presented. The black square highlights the area within Fano with the highest case density. In the top-right corner, a map of Italy indicates Fano municipality with a blue triangle.

Panel B. The geographic distribution of cases within the area of highest density is presented. The circles indicate the median distances from the epicentre by week of symptom onset.

Panel C. A boxplot showing the distances from the epicentre (defined as the centroid of geolocated cases from 12–25 August) for each week of symptom onset. Due to the small number of cases, data from the first 2 weeks of the outbreak were merged for the analysis. Number of cases: 12–25 Aug: n = 7; 26 Aug–1 Sep: n = 13; 2–8 Sep: n = 32; 9–15 Sep: n = 57, 16–22 Sep: n = 42, 23–29 Sep: n = 21; 30 Sep–6 Oct: n = 20; 7–13 Oct: n = 7.

## Entomological investigation and whole genome sequencing of *Aedes* and human samples

Trapping of mosquitoes was conducted over 9 non-consecutive days between 11 September and 10 October 2024, with each trapping session lasting 24 h (Figure 2). All insects were species identified, mosquitoes caught by the same trap at the same time were grouped into two pools and then tested for viral RNA with RT-PCR [10]. On 16 and 21 September, three mosquito pools of *Ae. albopictus* tested positive for dengue virus.

**Figure 3:**
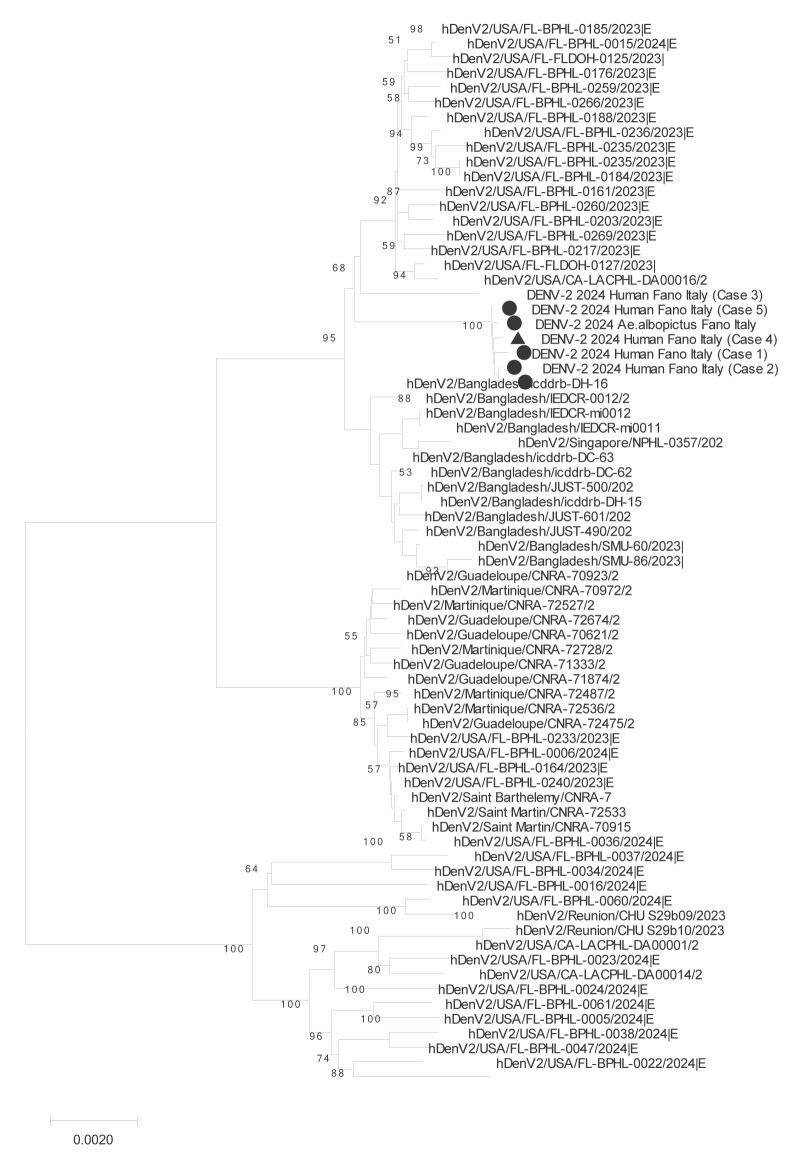
Neighbour-joining phylogenetic tree of dengue virus 2 isolates from human samples (n = 5) and mosquito pool (n = 1) in a dengue outbreak, Fano, Italy, August–October 2025, compared with global isolates^a^

^a^ More than 1,700 recent global DENV-2 isolates from the GISAID repository (https://gisaid.org/publish/) included.

Total RNA from two DENV-positive mosquito pools and 15 positive human samples underwent whole genome sequencing (WGS) following a SISPA protocol [11,12], library preparation by Illumina DNA Prep kit (Illumina Inc., San Diego, the United States (US)) and library enrichment using a capture probes panel for DENV serotypes 1–4 (Twist Bioscience, San Francisco, US). Deep sequencing was performed on the NextSeq2000 platform using NextSeq1000/2000 P1 Reagents (300 cycles) and 150 bp paired-end reads (Illumina). We obtained complete consensus sequences for one mosquito pool and five human samples. All sequences were nearly identical, with only rare single-point mutations in three sequences. Genotyping, performed using the Genome Detective typing tool (https://www.genomedetective.com/app/typingtool/dengue/), identified the strains from Fano as genotype II, lineage F1 [13]. A neighbour-Joining phylogenetic tree (MEGA11 software package, version 11.0.9, https://www.megasoftware.net/), based on over 1,700 recent global DENV-2 isolates from the GISAID repository (https://gisaid.org/publish/), revealed the highest similarity to isolates from the US (Florida and California) and Bangladesh (Figure 3). For more details see, Supplementary Material.

## Public health measures

Following the Italian National Plan for Prevention, Surveillance and Response to Arboviruses (2020–2025) [8], an initial mosquito-control treatment was performed targeting *Ae. albopictus* by using larvicides (methoprene pure-denatonium benzoate), adulticides (cypermethrin-tetramethrin) and removing breeding sites. The control measures were taken on 11–12 September within a radius of 200 m from the residence of the index case (Figure 1). As the outbreak spread, a second treatment was scheduled for 15–18 September, targeting the entire municipality of Fano and residential urban areas within a 2 km radius around Fano where cases had been identified. Due to adverse weather conditions on 17–18 September, the treatment was completed on 22–23 September. The latest treatment was conducted from 6 to 8 October. Entomological monitoring included BG-sentinel traps (Biogents, Regensburg, Germany) and gravid traps.

From 12 September, all blood donors who lived in or visited Fano and neighbouring municipalities epidemiologically linked to the outbreak were screened for DENV with PCR. By 28 October, over 2,200 donations were tested, all yielding negative results. On the same date, screening was extended to organs, tissues and haematopoietic stem cell donations across the province. No positive donations were detected.

During the outbreak, the Regional Operational Group for Health Emergencies (GORES), consisting of RHA, LHA, Civil Protection, experts in infectious diseases and virology, experts in public health risk communication and experts from Istituto Zooprofilattico Sperimentale Umbria-Marche (IZSUM) met five times to monitor the outbreak, issuing a press release after each meeting (Figure 1B, Figure 2). The public was advised on protective measures against mosquito bites, information was published on the LHA website [14]. Hospitals, physicians and pharmacies were alerted to be vigilant for potential dengue cases and notify them timely. Initially limited to Fano, the alert was extended to the entire province of Pesaro and Urbino on 20 September. The RHA and LHA consulted regularly the Italian National Health Institute (Istituto Superiore di Sanità (ISS)) and the Ministry of Health (MoH) to implement containment measures as outlined in the national arbovirus control plan [8]. From 1 October, dengue testing was offered to family and household members of cases. Approximately 30 samples were taken and all tested negative.

## Discussion

The outbreak of 199 autochthonous dengue cases in Fano marks a notable shift in the epidemiology of dengue in Europe. Our estimates suggest a higher transmissibility than previously observed for DENV in Italy [15], with levels comparable with transmission in tropical areas [16] and with previous chikungunya outbreaks in Italy [17,18]. The estimated reporting delays and their temporal changes are consistent with those observed in previous dengue outbreaks in Italy [15].

The outbreak was concentrated primarily in a residential neighbourhood with predominantly terraced houses of two floors and tree-covered private gardens, bordered by a wide green area and a partially dry canal. Although 50% of the weekly cases were within 800 m of the initially affected area, we cannot exclude the possibility of additional undetected hotspots either within or outside the Fano municipality. Active clinical, epidemiological, virological and entomological surveillance is ongoing to assess the full extent of this outbreak and detect possible secondary transmission in other Italian regions.

Our analyses were based on data notified until 28 October. By 20 November, six additional cases (four confirmed and two probable) have been notified, and we are awaiting laboratory results for two suspected cases. The symptom onset date of the most recent case was on 31 October. Typically, the duration required to close a dengue outbreak is around 45 days. Therefore, we still consider the outbreak ongoing.

Consistent with previous reports [15,17,18], the detection of the outbreak and the triggered control interventions led to a reduction in the reporting delay, a likely increase in public awareness and to a rapid decrease of transmissibility below the epidemic threshold within 2 weeks (approximately corresponding to one generation time).

As in previous dengue outbreaks in Italy, we could not find the primary source of the outbreak, for instance a viraemic traveller introducing the virus. Thus, the virus was likely already present in the community, which contributed to delays in reporting and undetected infections prior to the identification of the index case, after which the vector control measures, and active case-finding were implemented. Although transovarial transmission in the mosquitoes cannot be entirely excluded, current evidence does not substantiate this mechanism, particularly in temperate regions. Further microbiological analyses are required to explore possible connections with previously diagnosed cases in Italy.

A site visit by the MoH and the ISS was conducted on 28 October, to identify strengths and potential areas for improvement in future responses to similar outbreaks. The main barriers to early detection are low population awareness on travel-related dengue and lack of consideration of dengue as a cause of local fevers in late summer among physicians. In October 2024, MoH issued new testing criteria for DENV in Italy to include suspected cases with no travel history or epidemiological links to dengue cases. However, in Italy as well as in other European Union (EU) countries with ecologically favourable conditions for transmission, training of healthcare providers to consider dengue as a potential diagnosis, even in patients without travel history, is needed. Likewise, more investments should be made in the EU/European Economic Area (EEA) countries in increasing public awareness on dengue, emphasising the importance of early medical consultation when experiencing fever following travel.

Warmer temperatures and altered rainfall patterns create more favourable conditions for *Ae*. *albopictus*, the primary vector of dengue transmission in Italy and Europe [19]. Also considering 2023 data [20,21], this outbreak consolidates evidence that seasonal conditions can support efficient and sustained dengue transmission in Italy.

## Conclusion

*Aedes*-borne diseases like dengue should be considered among expected locally acquired seasonal diseases in EU countries with favourable seasonal ecological conditions for transmission. Public health professionals must adapt to this evolving epidemiology by improving prevention, preparedness and awareness to mitigate dengue transmission and its impact in Europe. The timely identification of cases, also without links to endemic countries, is a critical aspect to prioritise.

## Supplementary Data

1118000 Supplementary Material
