# Supplementary material for: Autochthonous dengue outbreak in Marche Region, Central Italy, August to October 2024
Source: Euro Surveill. 2024 Nov 21;29(47):2400713. doi: 10.2807/1560-7917.ES.2024.29.47.2400713 (PMC11583309; doi:10.2807/1560-7917.ES.2024.29.47.2400713)
Supplement: Supplementary Material [file 24-00713_SACCO_Supplement.pdf]

Supplementary Materials

Unexpectedly high transmissibility fuelling an autochthonous dengue outbreak in Marche Region, Central Italy, August-October 2024

This supplementary material is hosted by Eurosurveillance as supporting information alongside the article "Unexpectedly high transmissibility fuelling an autochthonous dengue outbreak in Marche Region, Central Italy, August-October 2024", on behalf of the authors, who remain responsible for the accuracy and appropriateness of the content. The same standards for ethics, copyright, attributions and permissions as for the article apply. Supplements are not edited by Eurosurveillance and the journal is not responsible for the maintenance of any links or email addresses provided therein.

Table of Contents

Case definition **2**
Estimation of the reproduction number **2**
Additional results and sensitivity analyses **3**
Statistical analysis of reporting delays **5**
Methods of laboratory investigation **5**
References **6**

Case definition

As for the case definition criteria of Italian National plan for prevention, surveillance and response to arboviruses (2020-2025) [1], a probable case is defined as an individual exhibiting symptoms consistent with dengue with a positive serology for immunoglobulin (Ig)M antibodies. A confirmed case requires laboratory confirmation, which may involve virus isolation, detection of viral RNA, or dengue viral antigen (NS1), or the presence of dengue-specific IgM antibodies in a single serum sample AND confirmation by neutralization, or seroconversion or four-fold antibody titre increase of dengue specific antibodies in paired serum samples.

Estimation of the reproduction number

To estimate R(t), we used the same methodology presented in [2,3,4]. We assumed that the daily number of new autochthonous dengue cases (by date of symptom onset) with infection acquired in Fano (PU, Italy), L(t), can be approximated by a Poisson distribution according to the equation

$$L(t) \sim \mathrm{Pois}\left(R(t)\sum_{s=0}^{t}\varphi(s)\,C(t-s)\right),$$

where

- $C(t)$ is the number of cases with symptom onset at time t residing in Fano (PU, Italy);
- $R(t)$ is the net reproduction number at time t;
- $\varphi(s)$ is the distribution of the generation time evaluated at time s, which is assumed to follow a Gamma distribution with mean 18.3 and standard deviation 8.1, as estimated in [4].

The likelihood L of the observed time series of cases from day 1 to day T conditional on $C(0)$ is thus given by

$$L = \prod_{t=1}^{T} P\left(C(t);\ R(t)\sum_{s=1}^{t}\varphi(s)\,C(t-s)\right),$$

where $P(k;\lambda)$ is the probability mass function of a Poisson distribution (i.e., the probability of observing k events if these events occur at a rate λ). The posterior distribution of R_t is estimated by using the MCMC Metropolis-Hastings sampling approach.

The posterior distribution of R_0 is estimated by applying the above-described procedure and by assuming that during the period where the epidemic showed exponential growth R_t=R_0. Specifically, by analyzing the log-transformed cumulative number of cases, we identified the exponential growth of cases as occurring between August 31 and September 13, 2024 (Figure S1).

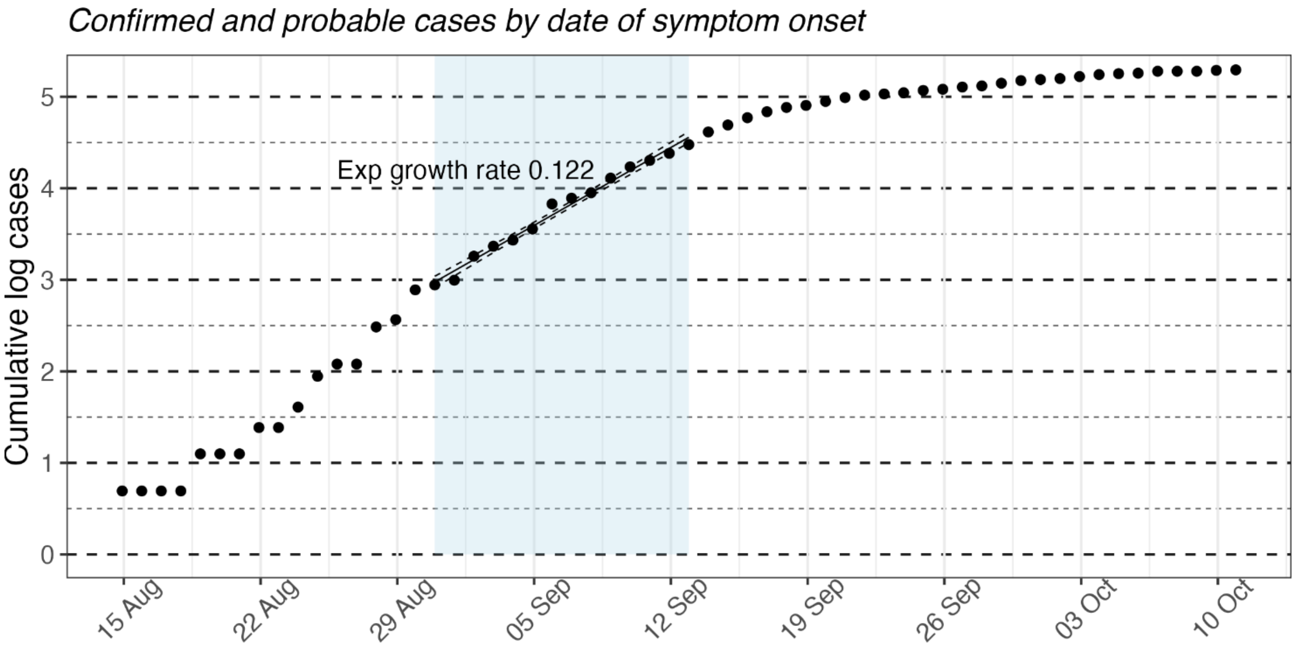

Figure S1. Identified time window (shaded area) of exponential growth in the cumulative number of cases for the estimation of R_0. On the x-axis the date, on the y-axis the log-transformed number of the cumulative number of daily cases by date of symptom onset. Points represent the log-transformed cumulative number of daily cases infected in Fano (PU, Italy). The solid line represents the regression slope fitted on the log-transformed cumulative number of cases; the dashed lines represent the 95% confidence interval.

Additional results and sensitivity analyses

In the baseline analysis, estimates of the reproduction number were obtained by considering all symptomatic cases ascertained in Fano (PU, Italy), including both lab-confirmed and suspected cases. Alternative estimates of the transmission patterns were explored by considering only cases confirmed via PCR. All the performed analyses provided consistent results on the estimated basic reproduction number (Table S1).

Table S1. Estimates of the basic reproduction number R_0 as obtained under different model assumptions.

	All symptomatic cases	***Symptomatic cases confirmed via PCR***
Period of exponential growth	31 August – 13 September	31 August – 13 September
Exponential growth rate	0.122 (95%CI: 0.112 – 0.131)	0.144 (95%CI: 0.132 – 0.157)
R_0	2.66 (95%CI: 2.08 - 3.31)	2.60 (95%CI: 1.93-3.36)

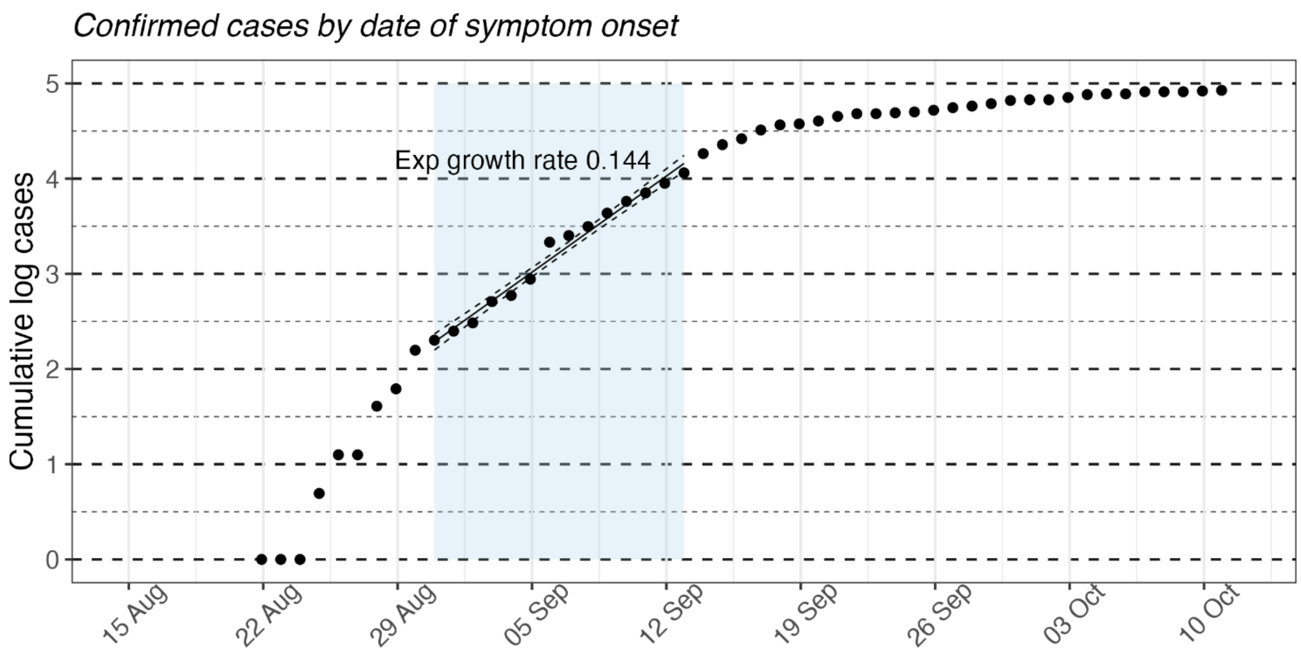

Figure S2. Identified time window (shaded area) of exponential growth in the cumulative number of confirmed cases for the estimation of R_0. On the x-axis the date, on the y-axis the log-transformed number of the cumulative number of daily confirmed cases by date of symptom onset. Points represent the log-transformed cumulative number of daily confirmed cases infected in Fano (PU, Italy). The solid line represents the regression slope fitted on the log-transformed cumulative number of confirmed cases; the dashed lines represent the 95% confidence interval.

The temporal dynamics of the net reproduction number R_t as estimated by considering only cases confirmed via PCR is close to the one presented in the baseline analysis (Figure S3).

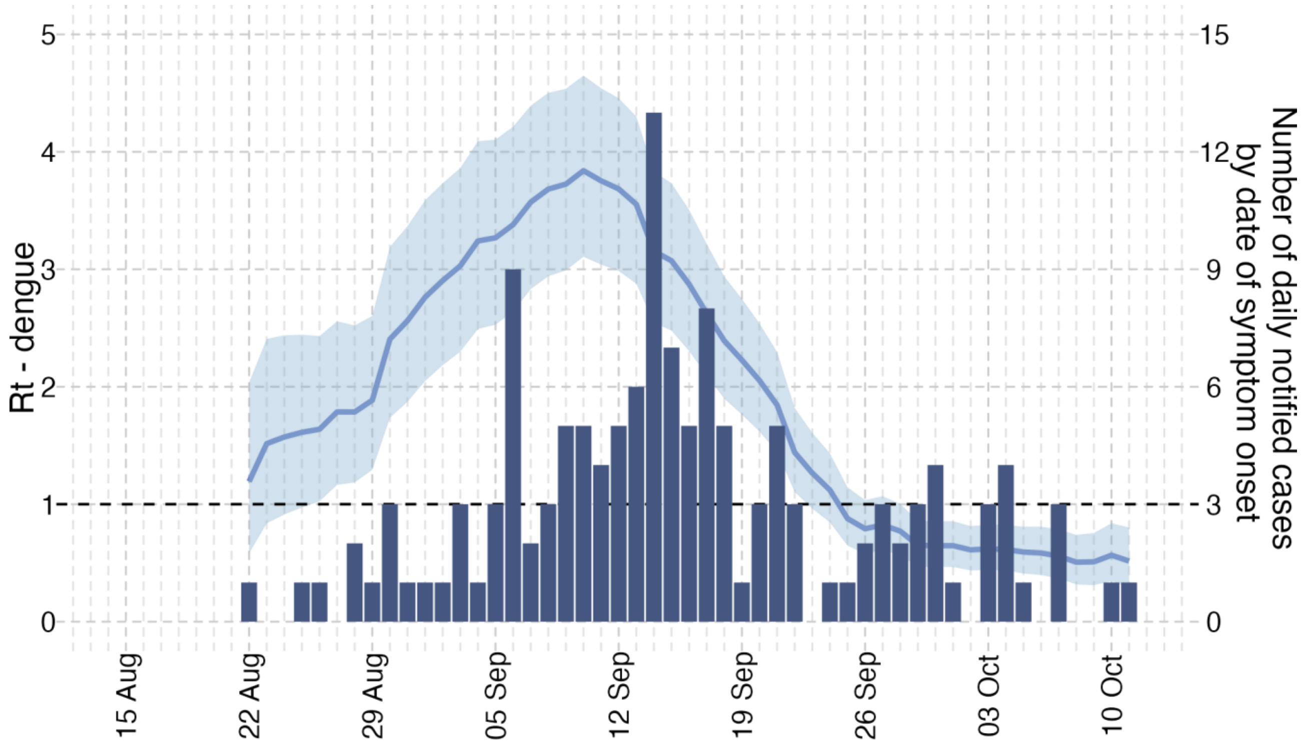

Figure S3. Temporal dynamics of the net reproduction number R_t as estimated by considering only cases confirmed via PCR. The solid blue line represents the estimated mean value of R_t; shaded area represents 95% credible interval. Bars represent the number of symptomatic cases confirmed via PCR by date of symptom onset (scale on the right y-axis).

Statistical analysis of reporting delays

We used a generalized linear regression model to investigate temporal changes in reporting delays. The reporting delay was defined as the time between symptom onset and notification to local health authorities. We assumed a Negative Binomial distribution for reporting delays and applied a generalized linear model (GLM) to the observed data. We considered one covariate defined as a qualitative grouping variable that classified cases in two groups: one including cases having symptom onset before outbreak detection (September 11) and one including cases having symptom onset afterwards. The GLM estimates are summarized in Table S2.

Table S2. Negative Binomial generalized linear model result.

	Model estimates (95% confidence interval)
Symptoms before outbreak detection	3.155 (3.017, 3.297)
Symptoms after outbreak detection	-0.855 (-1.038, -0.675)
Reduction (%)	57.5 (49.1, 64.6)

Methods of laboratory investigation

All blood samples were referred by the peripheral laboratories (mostly from Fano Hospital) to the regional reference center for dengue virus in Ancona (Virologia AOU delle Marche), or to the national reference laboratory for arboviruses in Rome (Istituto Superiore di Sanità). All samples were tested for positivity by RT-PCR (Dengue Serotyping, Clonit, Milan, Italy), performed on an automated platform (Ingenius, Elitech, Turin, Italy), or by DENV 1-4 CDC real time PCR [5]. All IgM and IgG serological assays were performed by commercial ELISA kits using an automated assay (Virclia IgG and IgM Monotest, Vircell, Granada, Spain), or by InBios Dengue Detect™ IgM Capture ELISA (FDA) (Seattle, WA, USA). Dengue virus NS1 antigen was detected using a commercial antigen-capture ELISA system (Bio-Rad Platelia™ Dengue NS1 Ag, Milan, Italy). Plaque Reduction Neutralization Test (PRNT) was performed as previously described [6]
Of 138 confirmed cases, 135 were positive by real time RT-PCR (in plasma and/or whole blood and/or urine) and/or for NS1 gene, whereas in three cases specific IgM antibodies were detected and confirmed by neutralization test. Positive serology (anti-DENV specific IgM and IgG ELISA antibodies) in RT-PCR negative patients retrospectively identified 61 probable cases. Real-Time PCR identified DENV-2.
The envelope gene from 57 patients was Sanger sequenced (Applied Biosystems, Whaltham, MA, USA). Figure S4 shown the results of the phylogenetic tree based on the envelope gene.
Sequencing analysis of the entire envelope coding sequence (envelope gene) was performed by Sanger sequencing on the ABI prism 3130 platform (Applied Biosystems, Whaltham, MA, USA).
Positive mosquito pools were sent to the CESME laboratories of the IZSAM for diagnostic confirmation. As part of the PNRR project INF-ACT (research node 4), DENV positive urine samples were sent by ISS to the GENPAT laboratories of IZSAM.
Total RNA purified from mosquitos and human samples were used for the assessment of WGS workflow that includes SISPA protocol, library preparation by Illumina DNA Prep kit (Illumina Inc., San Diego, CA) and library enrichment by a capture probes kit designed ad hoc for DENV 1-4 (Twist Bioscience, San Francisco, CA). Deep sequencing was performed on the NextSeq2000 using NextSeq 1000/2000 P1 Reagents (300 Cycles) and standard 150 bp paired-end reads (Illumina Inc., San Diego, CA).
Genotyping was performed using the genome detective typing tool (https://www.genomedetective.com/app/typingtool/dengue/). A Neighbour-Joining phylogenetic tree was constructed using MEGA11 software package, based on isolates from the GISAID repository (https://gisaid.org/)

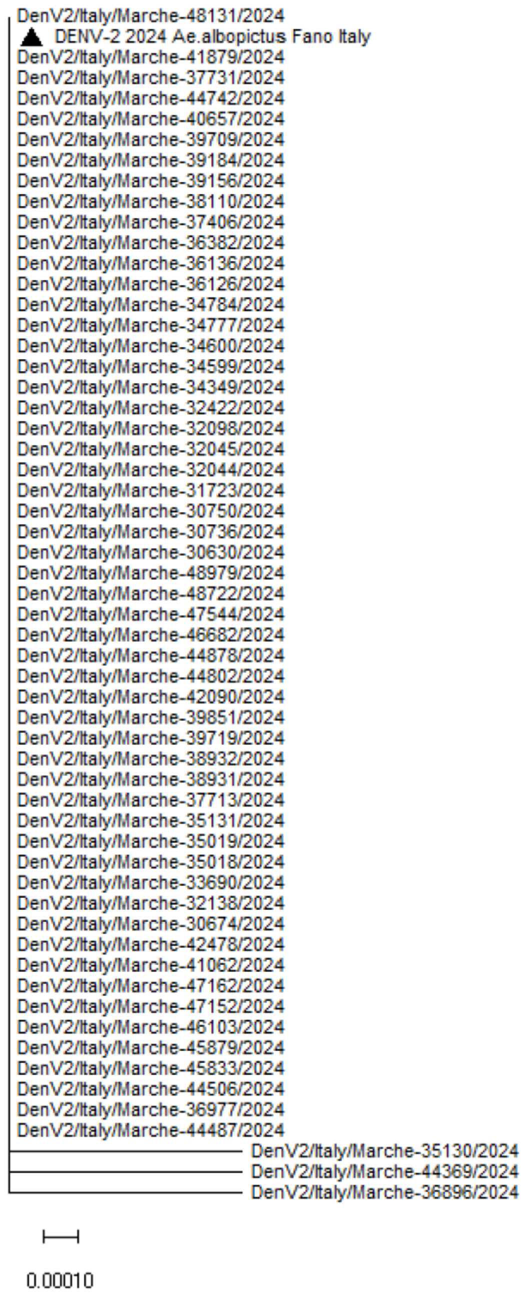

Figure S4. Phylogenetic tree of the envelope gene constructed using sequences from 57 human samples and one mosquito sample (indicated by a triangle).

References

1. Italian Ministry of Health. Piano Nazionale di prevenzione, sorveglianza e risposta alle Arbovirosi (PNA) 2020-2025. [National plan for prevention, surveillance and response to arboviruses (2020-2025)]. Rome: Ministry of Health; 2019. Italian. Available from: https://www.salute.gov.it/imgs/C_17_pubblicazioni_2947_allegato.pdf

2. Cori A, Ferguson NM, Fraser C, Cauchemez S. A New Framework and Software to Estimate Time-Varying Reproduction Numbers During Epidemics. American Journal of Epidemiology. 2013 Nov 1;178(9):1505–12.

3. Manica, M. et al. Transmission dynamics of the ongoing chikungunya outbreak in Central Italy: from coastal areas to the metropolitan city of Rome, summer 2017. Eurosurveillance 22, (2017).

4. Rovida, F. et al. The 2023 Dengue Outbreak in Lombardy, Italy: A One-Health Perspective. Preprint at https://doi.org/10.48550/ARXIV.2408.04768 (2024).

5. Centers for Disease Control and Prevention. **CDC DENV-1-4 Real-Time RT-PCR Multiplex Assay**. Available at: https://www.cdc.gov/dengue/hcp/diagnosis-testing/cdc-denv-1-4-real-time-rt-pcr-multiplex-assay.html. Accessed 21/10/2024.

6. Merakou, C.; Amendola, A.; Fortuna, C.; Marsili, G.; Fiorentini, C.; Argentini, C.; Benedetti, E.; Rezza, G.; Maraglino, F.; Del Manso, M.; et al. Diagnosis of Imported Dengue and Zika Virus Infections in Italy from November 2015 to November 2022: Laboratory Surveillance Data from a National Reference Laboratory. *Viruses* **2024**, *16*, 50. https://doi.org/10.3390/v16010050
